# Nanotechnology meets medicine: applications of atomic force microscopy in disease

**DOI:** 10.1007/s12551-025-01306-w

**Published:** 2025-04-03

**Authors:** Zita Matias, Catarina S. Lopes, Nuno C. Santos, Filomena A. Carvalho

**Affiliations:** 1 Faculdade de Medicina, Universidade de Lisboa, Avenida Professor Egas Moniz, 1649-028 Lisbon, Portugal; 2 ULSLO – Unidade Local de Saúde Lisboa Ocidental, Lisbon, Portugal; 3 GIMM – Gulbenkian Institute for Molecular Medicine, Av. Prof. Egas Moniz, 1649-035 Lisbon, Portugal

**Keywords:** Atomic force microscopy, Infectious diseases, Cardiovascular diseases, Cancer, Neurodegenerative diseases

## Abstract

Atomic force microscopy (AFM) is a scanning imaging technique able to work at the nanoscale. It uses a cantilever with a tip to move across samples’ surface and a laser to measure the cantilever bending, enabling the assessment of interaction forces between tip and sample and creating a three-dimensional visual representation of its surface. AFM has been gaining notoriety in the biomedical field due to its high-resolution images, as well as due to its ability to measure the inter- and intramolecular interaction forces involved in the pathophysiology of many diseases. Here, we highlight some of the current applications of AFM in the biomedical field. First, a brief overview of the AFM technique is presented. This theoretical framework is then used to link AFM to its novel translational applications, handling broad clinical questions in different areas, such as infectious diseases, cardiovascular diseases, cancer, and neurodegenerative diseases. Morphological and nanomechanical characteristics such as cell height, volume, stiffness, and adhesion forces may serve as novel parameters used to tailor patient care through nanodiagnostics, individualized risk stratification, and therapeutic monitoring. Despite an increasing development of AFM biomedical research with patient cells, showing its unique capabilities in terms of resolution, speed, and accuracy, there is a notable need for applied AFM research in clinical settings. More translational research with AFM may provide new grounds for the valuable collaboration between biomedical researchers and healthcare professionals.

## Introduction

The medical field has kept evolving, partly due to new technical developments, such as innovative imaging methods, which have provided more precise and faster diagnoses, as well as better quantitative parameters for risk stratification and therapeutic monitoring. Some noteworthy examples include at the macro-level X-ray imaging, computerized tomography, and magnetic resonance (Bercovich and Javitt 2018), and at the micro-level optical microscopy (Chen et al. 2011). However, with the increasing burden of chronic diseases such as cancer and cardiovascular diseases (World Health Organization 2022a) and emergence of new infectious diseases (Baker et al. 2022), early diagnosis and more personalized monitoring became more relevant. These diseases are frequently associated to changes in cells’ morphology, biomechanical properties, adhesion, and interaction forces with other components, and thus in function, motility and division (Gavara 2017).

Atomic force microscopy (AFM) is included in a group of techniques named scanning probe microscopy (SPM) techniques, which are based on local mechanical interactions between a probe and the sample surface (Sumbul and Rico 2019). These techniques lead to better resolution than conventional optical microscopy techniques’ diffraction limit, without most of the limitations of electron microscopy (Santos and Castanho 2004; Sumbul and Rico 2019). SPM evolved from its first iteration, the invention of scanning tunneling microscopy (STM), in 1982, which uses the exponential decay of the tunneling current between a metallic probe and a conductive surface as a function of the increasing distance to create its topographical images (Santos and Castanho 2004; Sumbul and Rico 2019). Despite the unprecedented resolution, this technique required its samples to have electrical conductivity (not common in biological samples) and imaging to be done in vacuum (Sumbul and Rico 2019). AFM represented the next step for SPM upon its invention by Gerd Binnig, Calvin Quate, and Christoph Gerber, in 1986 (Binnig et al. 1986). Since then, AFM, initially more focused in other areas such as material sciences (Nguyen-Tri et al. 2020), became progressively more important for the study of biomolecules such as proteins and nucleic acids, and then for living cells in aqueous environment (Uchihashi and Ganser 2020). This review intends to show how the AFM technique has been used and how biomedical applications are continuing to pave the way for using AFM in a clinical setting.

## AFM technique

There are three main components in conventional AFM: a detector, a controller, and a data or image processing system (Pisano and Gilson 2019). The AFM sample is assembled on a piezoelectric support, with the possibility of being imaged in vacuum, air (or other gas), or liquid environment. In the detection system, the surface of the sample is scanned by a probe within an AFM head through parallel lines. The scanning can be done through moving the probe or the sample itself through the piezo scanner’s movement on the z-axis, while it is scanned on the x–y axis of the support (Eaton et al. 2019). The tip of the probe is located under a flexible cantilever, which allows for signal transduction, indirectly measuring the local interaction force between the sample and those of the scanning tip since the cantilever will bend with the attractive and repulsive forces between the tip and the sample (Carvalho et al. 2015). These deflections are detected by using a laser pointed towards the extremity of the cantilever (on the surface opposite to the tip), whose reflected light when hitting a four-quadrant photodiode detects the changing positions of the cantilever extremity (Gavara 2017). While the cantilever is not deflected (usually when resting away from the sample), the photodiode is positioned such that the laser is equally split between the top and bottom quadrants. When the tip scans a change in height of the tip over the sample, there is a change in the interaction force. The change in deflection of the cantilever causes a change in the proportion of the reflected laser that hits the top and bottom quadrants and thus a difference in photovoltage between these quadrants. This deflection is used for the feedback mechanism for real-time adjustment of the vertical position of the cantilever in relation to the sample to keep the deflection constant (if in contact mode) (Gavara 2017).

**Fig. 1:**
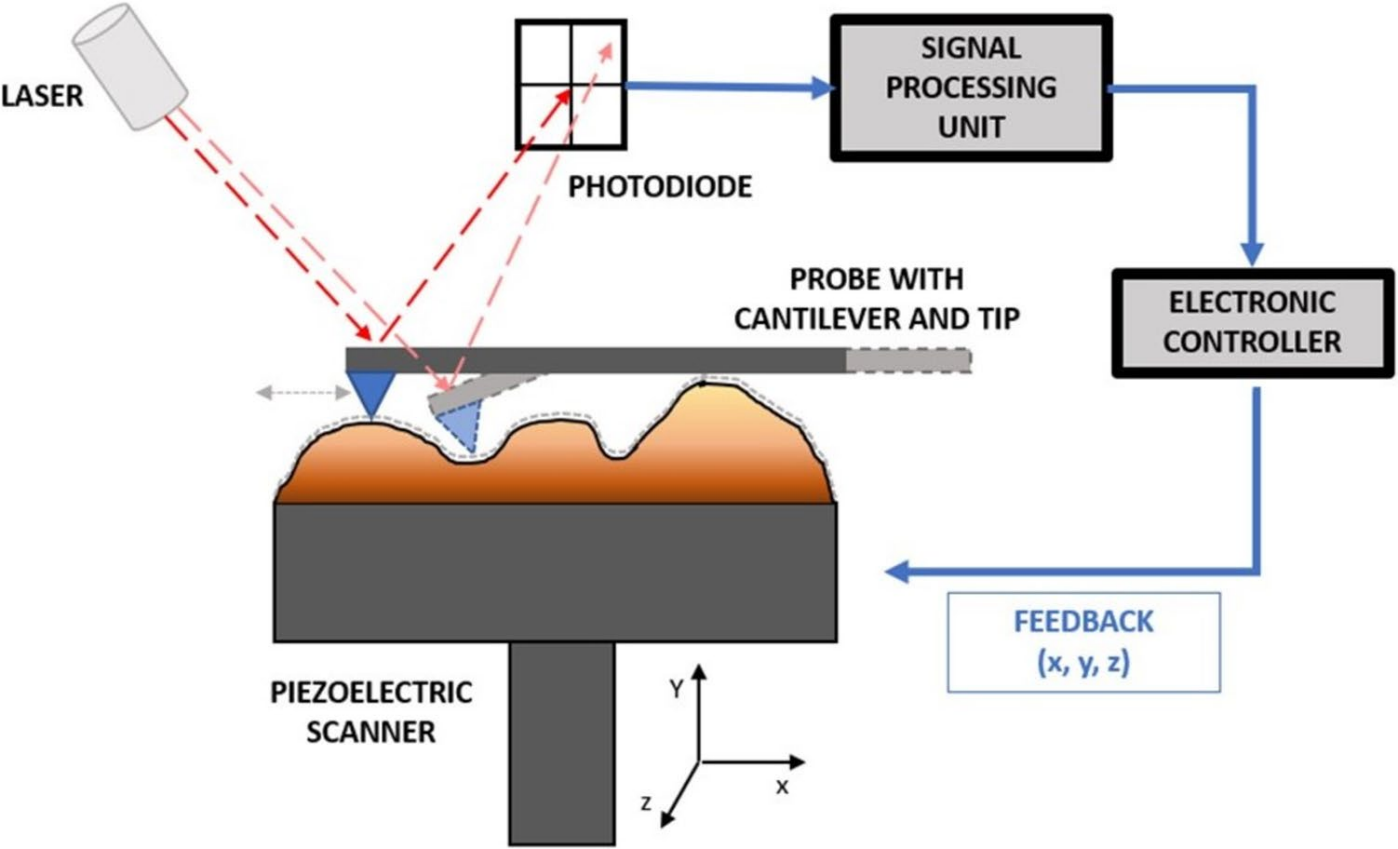
Schematic diagram depicting a typical AFM system. Adapted from (Ishida and Craig 2019)

In the data processing system, the measurement of the interaction force is recorded for each position using an electronic controller and computer. The values for the interaction forces are then used for the reconstitution of a three-dimensional image of the sample (Gavara 2017). These AFM components are shown in a schematic representation image (Fig. 1).

This technique allows for the detection of forces as small as 7–10 pN and can produce images with a lateral resolution of 0.5–1nm and axial resolution of 0.1–0.2 nm (Nikitaev et al. 2021; Sullan et al. 2013). This allows for the imaging of single molecules or even single atoms (Gross et al. 2009). In contrast, conventional optical microscopy is mostly restricted by the optical diffraction, making it difficult to go below 200–300 nm, rendering it impossible or, at least, difficult to detect single molecules or their clusters (Prakash et al. 2021).

Some of the advantages of AFM relative to other microscopy techniques are its simple sample preparation, ability to image biological samples in physiological conditions, and ability to easily identify and rectify artifacts of the tip and scanner due to the system’s high magnification and sensitivity. However, artifacts can be created by the alterations induced during the tip-sample contact and, for biomedical researchers, lack of standardized criteria for comparing results from different AFM studies assessing cell morphology and biomechanical properties (Zhu et al. 2022). AFM is also highly advantageous due to its high sensitivity in force detection and high-resolution imaging (Eaton et al. 2019). Previous limitations in scanning speed have been overcame through high-speed AFM (HS-AFM) for faster image throughput and imaging of live processes (Pi and Cai 2019). However, HS-AFM still presents some limitations: the tip may affect the processes in the sample, it requires the sample to be immobilized on a substrate, restricting the dynamics and natural interaction between biomolecules, and it has a small scanning area of approximately 1 μm^2^, limiting the observation of larger scale processes (Ando 2013; Casuso et al. 2020; Yu and Yoshimura 2021).

An important limitation of AFM use in biomedical research is its demanding requirements of sample preparation for live cell imaging under physiological conditions. To avoid cell deformation by the tip, some researchers prefer to fix cells using chemical agents, despite the fact that cells may become stiffer and that this process may itself cause some artifacts (Liu et al*.* 2012). Furthermore, to maintain cells alive during imaging, several factors in their environment need to be controlled, such as using a medium with nutrients for cell culture and a chamber with specific atmosphere and temperature conditions (Cho et al*.* 2023). Even so, cells must be carefully monitored during AFM imaging, in order to check their survival and attachment. When cells are accidentally detached by the AFM tip, their assessment can be continued by finding an intact section of cells or else, the whole cell sample must be prepared again (Cho et al*.* 2023).

AFM has different scanning modes according to different situations. The most commonly used modes for imaging are contact mode and tapping (intermittent contact) mode (Pi and Cai 2019). In contact mode, the tip remains in contact with the sample’s surface, generating strong repulsion forces between tip and sample (Pi and Cai 2019). In tapping mode, the cantilever is constantly oscillating. As this reduces the interaction between tip and sample, it frequently minimizes the damages in soft samples (Pi and Cai 2019). This potentially leads to higher accuracy in sample images and is important for HS-AFM studies.

Besides topographical imaging, another main application of AFM is single-molecule force spectroscopy (SMFS) at certain locations of the sample, using the tip as indenter (Gavara 2017). The cantilever behaves as a spring that obeys Hooke’s law when the tip applies force onto the sample, causing the sample to deform. Forces (F) acting on the cantilever tip can be calculated using the pre-determined spring constant of the cantilever (K), related with its stiffness, and the cantilever deflection (d) measured, using the formula F = Kd (Gavara 2017). During the measurement, the vertical displacement of the cantilever is also recorded and later used to generate force *vs.* displacement curves (Gavara 2017). SMFS can be adapted for (bio)chemical specificity by attaching a specific molecule to the tip (e.g., antibody, other protein, peptide, or small molecule) and scanning the surface in order to a find an antigen or other specific ligand (Patel and Kranz 2018).

The mechanical properties of cells, such as their stiffness, may change in pathological conditions. This can be probed by applying a known force on the sample and measuring the resulting deformation (Gavara 2017). By relating the force and deformation, the stiffness of the sample can be estimated, frequently quantified as Young’s modulus, *E* (Gavara 2017). *E* is a parameter included in the Hertz model for mechanical characterization that serves as a measure of a solid’s stiffness or resistance to elastic deformation under load (Narayanaswamy et al. 2019). The underlying principle is when a material undergoes deformation as a load causes it to be compressed or extended, returning to its original shape when the load is removed (Narayanaswamy et al. 2019). Stiffer materials undergo less deformation, leading to a higher *E* value (Narayanaswamy et al. 2019).

Other imaging techniques, such as optical microscopy, fluorescence microscopy, and infrared (IR) spectroscopy, can be combined with AFM for their microscale imaging and can be used to provide complementary information (Zhou et al. 2017).

A specific example has been the use of microelectrode arrays and calcium imaging to correlate the biophysical properties of cardiomyocytes obtained by AFM with the cells’ electrical activity and calcium ion movements during its action potential, respectively. These have been useful for studying complex cardiac events including aminophylline-induced arrythmias and alterations in contractility induced by lamin mutations (Borin et al. 2020; Caluori et al. 2019; Klimovic et al*.* 2022; Marimon et al*.* 2025; Tian et al*.* 2017).

## Clinical applications

The AFM assessment of cell topography, force spectroscopy and/or quantitative imaging may be used to distinguish normal from diseased cells, in parameters such as cell morphology, cell adhesion, and biomechanical behavior (Gavara 2017). This can enhance the understanding of the disease status of cells, but many studies also highlight AFM as a potential candidate for investigating emerging diseases and, in known diseases, for earlier diagnosis, risk stratification, therapeutic drug monitoring and for finding therapeutic drug targets or prognostic biomarkers (Krawczyk-Wołoszyn et al. 2024). A significant example is the use of human embryonic-stem cell derived organoids to facilitate trials of new technologies and drugs at the cellular level, including model retinal-secreted extracellular vesicles or drug delivery and vascular explants for better grafts (Adelson et al*.* 2021; Arthur et al*.* 2023).

### Infectious diseases

It has been shown that microorganisms responsible for many infectious diseases are constantly evolving to resist game-changing milestones in treatment like antimicrobial drugs and vaccines. This section summarizes examples of AFM being used to investigate the pathophysiology and potential therapeutic targets in diseases with significant morbidity and mortality worldwide caused by viruses, bacteria, fungi, and parasites.

#### COVID-19

Starting from late 2019, the world was faced with an emerging pandemic originating from a previously unknown coronavirus, requiring much research to characterize its structure, transmission, and potential therapies, including vaccines. Coronavirus disease 2019 (COVID-19) was initially characterized as a respiratory viral illness, later discovered to induce a generalized hyperinflammatory response, leading to multisystemic manifestations with great morbidity and mortality, particularly in vulnerable populations (Silva et al*.* 2023). Its etiological agent, severe acute respiratory syndrome coronavirus 2 (SARS-CoV-2) is an enveloped single-stranded ( +) RNA virus (Lyonnais et al. 2021). AFM was used in research of this new pathogen to visualize its structure and quantify the virus’ interaction with cells and abiotic surfaces.

**Fig. 2:**
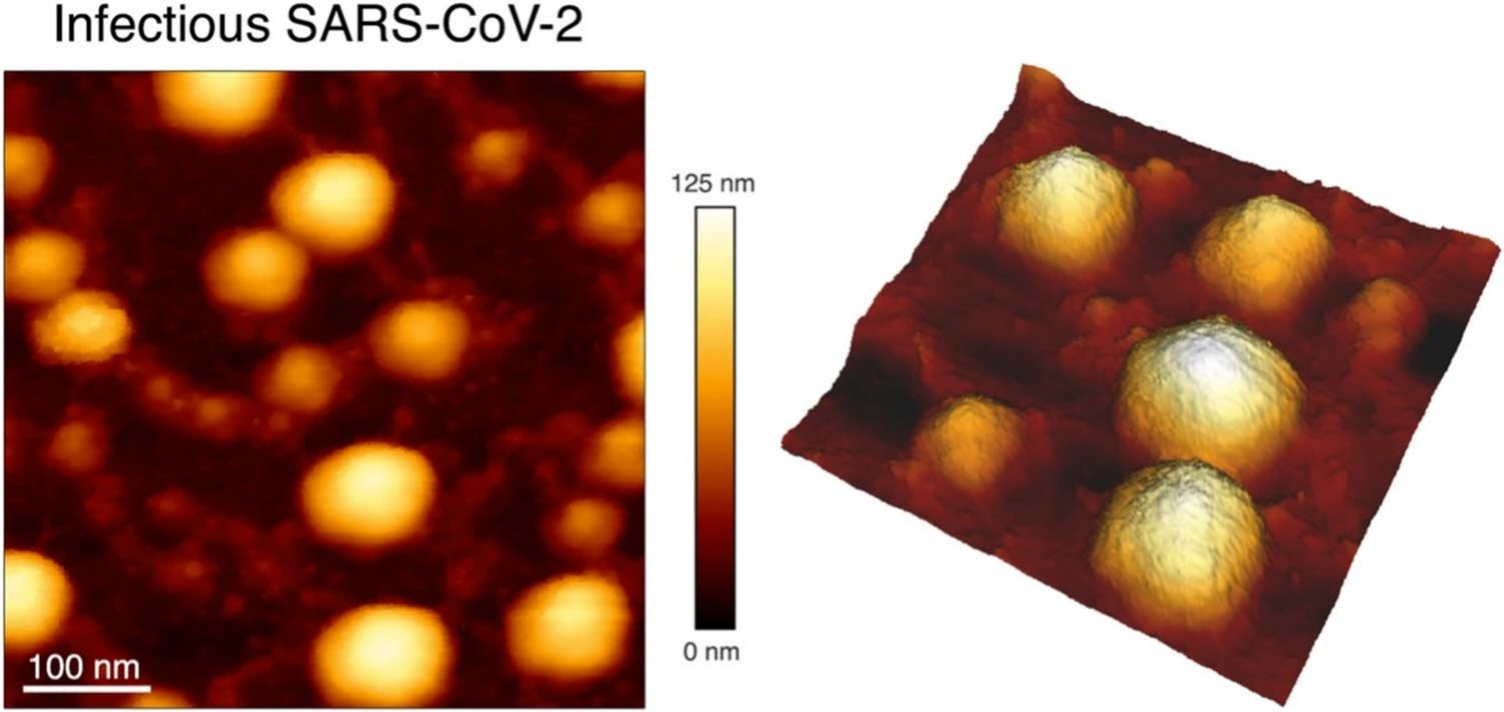
Native infectious SARS-CoV-2 virions imaged by AFM. Topographic image and 3D projection of native infectious SARS-CoV-2 virions adsorbed on a poly-l-lysine-coated mica surface using quantitative imaging mode AFM in buffer (adapted from (Lyonnais et al. 2021), with permission)

AFM was used initially to characterize the overall morphology of SARS-CoV-2, yielding images of particles with a crown of increased stiffness, shown in Fig. 2 (Lyonnais et al. 2021), as well as its spike protein that mediate host recognition and membrane fusion (Cardoso-Lima et al. 2021; Lim et al. 2021). HS-AFM was used for real-time visualization of its interaction with the human angiotensin-converting enzyme 2 (hACE2) receptor (Lim et al. 2021). These important functions of the spike protein make it an important therapeutic target, such as for binding inhibitor peptides to prevent viral entry, in addition to its relevance for vaccine development (Yang et al. 2020).

An important way to limit the spread of SARS-CoV-2 is its inactivation on abiotic surfaces, through chemicals, heat or ultraviolet radiation (Lyonnais et al. 2021; Xue et al*.* 2024; Tomás et al. 2022). Xue et al. used AFM to show the effects of ultraviolet germicide irradiation on viruses, namely showing that despite apparently remaining structurally intact, they had lost the ability to replicate normally (Xue et al. 2024).

AFM was also explored as a potential complementary tool to assess novel and faster detection methods for SARS-CoV-2, when currently a real-time reverse transcription polymerase chain reaction (RT-PCR) is used to establish its diagnosis (Dutta et al. 2022). As an example, Kurmangali et al. aimed to develop an electrochemical impedance spectroscopy (EIS)-based aptasensor employing an interdigitated gold electrode (IDE) to detect the SARS-CoV-2 spike protein and its whole viral particle (Kurmangali et al. 2022). AFM imaging was used to correlate the changes in the morphology of the virus and its respective spike protein with its immobilization and entrapment by the biosensor (Kurmangali et al. 2022).

#### AIDS

Despite the development of chronic medical treatment and prevention therapy with great benefits in quality of life and longevity, acquired immunodeficiency syndrome (AIDS) still remains prominent on global disease burden, with an estimated 38.4 million people living with human immunodeficiency virus (HIV), its pathogen, worldwide in 2021 (World Health Organization 2023c). HIV infection, at an initial phase, may have mild unspecific symptoms, later progressing towards AIDS-defining conditions such as candidiasis of the pulmonary tract, tuberculosis co-infection, and Kaposi sarcoma (Yoshimura, 2017). HIV-1, specifically, is an enveloped lentivirus presenting in its membrane the viral envelope glycoprotein complex gp120/gp41, surrounding its viral core (Rousso and Deshpande 2022). This viral core contains two copies of genomic RNA as well as the enzymes reverse transcriptase and integrase (Rousso and Deshpande 2022). The study of its pathophysiology and possible therapeutic targets has been frequently centered on points in the process where the virus is vulnerable to loss of structural integrity and failure, namely viral entry, maturation, uncoating, and budding (Rousso and Deshpande 2022).

HIV-1 viral membrane proteins such as gp120 and viral capsid proteins such as p24 can serve as identifying new biomarkers for HIV-1 in diagnostic techniques besides the current gold standard nucleic acid tests (Centers for Disease Control and Prevention 2023; World Health Organization 2021b). AFM was used to validate novel biosensors for HIV-1 using these biomarkers, including flexibility for wearable device application (Shin et al. 2019; Islam et al. 2019).

Viral membrane proteins are also important for mediating viral core entry into the host cell via membrane fusion (Rousso and Deshpande 2022). Blocking this step of viral entry is a significant therapeutic target for current therapies and for the development of new ones. AFM was used to visualize and assess the effect of membrane-active virucidal agents and fusion inhibitor molecules such as T-1249 and enfuvirtide (Franquelim et al. 2013; Huarte et al. 2016). Novel delivery systems for antivirals were also assessed using AFM. Namely, Srivastava et al*.* developed a delivery system for amprenavir using carbon nanotubes, showing its advantages with a sustained drug release and reduced toxicity (Srivastava et al*.* 2024).

The uncoating of the viral core and dismantling of the RNA capsid is required for the reverse transcription of HIV-1 viral genome and integration with the host chromosomal DNA, making this an interesting target for novel therapeutics. Some AFM studies assessed capsid stability and remodeling during uncoating (Ramalho et al. 2016; Rankovic et al. 2017). They examined the effect of mutations that increase the stiffness of the RNA capsid, which would impair uncoating, and were shown to decrease infectivity, therefore displaying potential as an anti-HIV strategy.

#### Tuberculosis

Tuberculosis is a disease of significant global burden: in 2023, an estimated 10.8 million people fell ill with tuberculosis, including both its pulmonary form and extra-pulmonary manifestations (World Health Organization 2024c). Its pathogen, *Mycobacterium tuberculosis*, is a rod-shaped, non-spore-forming, thin aerobic, and acid-fast bacterium (Raviglione 2018). *Mycobacterium tuberculosis* is challenging to eradicate in patients because it can survive in adverse conditions, prompting research with AFM to evaluate its morphological adaptations according to environment in terms of drug resistance and latency.

Farnia et al. studied the differences in morphology between susceptible and drug resistant strains of *M*. *tuberculosis* (Farnia et al. 2010). It was shown that highly drug-resistant strains had a greater proportion of bacteria with a round shape (cocci), unlike the rod shape seen in susceptible strains, even when placed in favorable conditions. It was suggested that these cocci forms attach more firmly to the surface and help to distribute the effects of environmental hazards across more individuals, increasing the likelihood of their overall survival (Farnia et al. 2010). More recently, different types of pili in *M. tuberculosis* were identified and correlated with susceptible and drug-resistant strains (Farnia et al. 2023). These differences in drug susceptibility could be translated into new and faster approaches for antibiotic susceptibility tests (Vocat et al. 2023).

Other authors studied *M*. *tuberculosis* under hypoxic conditions, simulating its presence within a granuloma (Jakkala and Ajitkumar 2019). This study showed that hypoxic *M*. *tuberculosis* had an increased surface roughness and thickness, which contributes towards preventing the entry of antitubercular drugs such as rifampicin (Jakkala and Ajitkumar 2019). Velayati et al. also studied *M*. *tuberculosis* under hypoxic conditions (Velayati et al. 2011). They used AFM to track morphological changes over 48 months, showing that the bacilli were able to induce active tuberculosis in the peritoneal cavity of mice after 12 weeks (Velayati et al. 2011).

Due to the difficulty in detecting *M*. *tuberculosis* in latent states, including in genitourinary tuberculosis, AFM also contributed to the assessment of novel detection methods focused on antibody and DNA sequence tests (Costa et al. 2014; Sypabekova et al. 2019; Kamra et al. 2023).

AFM studies also identified potential new therapeutic targets for antitubercular strategies, including mechanisms of antibiotic resistance and important metabolic enzymes (Nautiyal et al. 2014; Perera et al. 2013). For example, Kataria et al*.* studied the structural details of the metabolic enzyme L-asparaginase as a target and tested active-site specific inhibitors in ex vivo conditions (Kataria et al. 2019). AFM images showed that the treated *M*. *tuberculosis* bacilli lost cellular morphology, indicating cell death (Kataria et al. 2019).

#### Candidiasis

*Candida albicans* is a pathogen present in human infections that can carry a high degree of morbidity, ranging from superficial oral or vulvovaginal candidiasis to life-threatening infections in immunocompromised patients. *Candida albicans* is a dimorphic fungus species, able to grow in either yeast or filamentous form (either pseudohyphae or hyphae) (Kadosh and Mundodi 2020).

A significant focus of study in *C. albicans* has been towards its cell wall, due to its functions in immune recognition and as a therapeutic target. A cell wall component, β−1,3-d-glucan, has been particularly identified through SMFS experiments as a moiety for immune recognition and a target for new treatments, such as synthesis inhibitors (including caspofungin) and the development of selective antibodies, with promising results (Couttenier et al. 2022; Formosa et al. 2013; Hasim et al. 2016; Quilès et al. 2017; Vanzolini et al. 2023). Other targets identified in the *C. albicans* cell wall using AFM-based SMFS are proteins that promote cell wall recognition by immune effector cells and inhibition of antifungal resistance proteins like Smi1 (Li et al. 2018a, b; Martin-Yken et al. 2018; Te Riet et al. 2017).

Another feature of *C. albicans* is its ability to form biofilms, including forming synergistic biofilms with bacteria, increasing the difficulty of eradication for each specie. This process has been studied in dental health and the colonization of endotracheal tubes, focusing on the effects of biofilm formation with bacteria from the oral flora and relevant towards tooth decay (Danin et al. 2015; Demuyser et al. 2014; Hwang et al. 2017; Jiang et al. 2022; Ovchinnikova et al. 2013; Peters et al. 2012; Wan et al. 2021). This influenced subsequent studies for design of biomedical devices in oral health: the adhesion of *C. albicans* to abiotic surfaces has been observed and the development of antifungal materials has been developed for general use, dentures and crown fillings, and filastatin coating for biomaterials (Aguayo et al. 2017; Klemm et al. 2021; Ozel et al. 2017; Real et al. 2013; Vargas-Blanco et al. 2017; Wen et al. 2016).

Several studies focused on therapeutic targets against *C. albicans*, including silver nanoparticles (Khalil et al. 2022; Silva Viana et al. 2020), antifungal-loaded nanoparticles (del Rocío Lara-Sánchez et al. 2024; Teixeira et al. 2023), photodynamic/photothermal inactivation (Baptista et al. 2017; dos Santos et al. 2016), and antimicrobial peptides (Bellavita et al. 2022; de Aguiar et al. 2020; Gonçalves et al. 2017). Notably, Dias et al. showed the synergic effect of fluconazole and the antimicrobial peptide ToAP2 on inhibiting biofilm viability compared to individual treatments through structural analysis using AFM (do Nascimento Dias et al*.* 2024).

#### Malaria

**Fig. 3:**
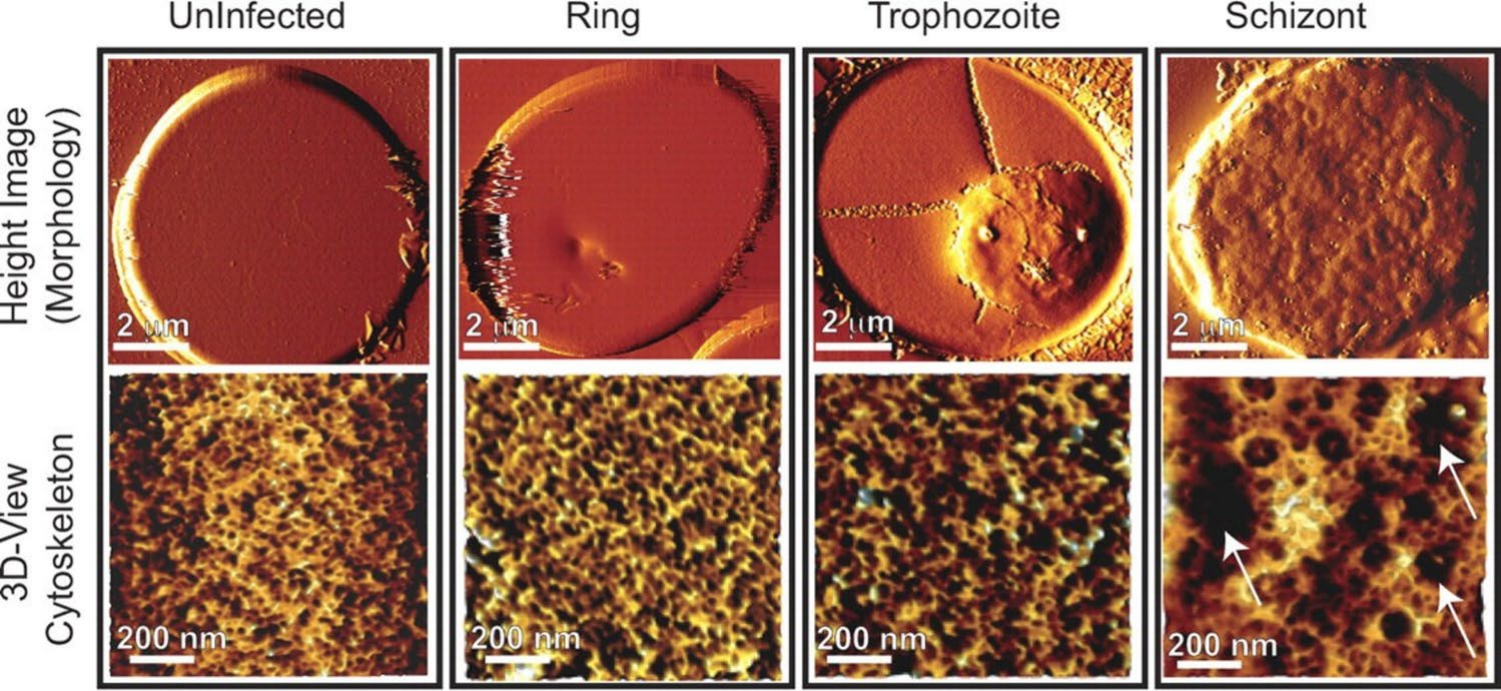
AFM imaging demonstrates progressive cytoskeletal changes on parasite development and merozoite egress. Uninfected erythrocytes and ring (10 h post-infection, hpi), trophozoite (25 hpi), or schizont (40 hpi)-infected erythrocytes were imaged using AFM. Processed images showed changes on the pRBC surface (mainly knob formation) by the trophozoite stage, at 25 hpi (upper panel). Extraction of cytoskeletal information (lower panel) from AFM images showed an increase in the mesh size of the spectrin network by the trophozoite stage, at 25 hpi, which expanded further by the schizont stage, at 40 hpi (adapted from (Millholland et al. 2011), with permission)

Malaria is one of the most important parasitic diseases in the world: in 2023, there were an estimated 263 million cases of malaria worldwide (World Health Organization 2024b). Malaria may manifest initially as cyclical fevers, with more significant complications eventually leading to severe anemia, cerebral malaria or death (Phillips et al. 2017). *Plasmodium falciparum* is one of the pathogens of malaria: this parasite is transmitted by the bite of infected female mosquitos and infects human cells, namely erythrocytes and hepatocytes, resulting in changes to its host cell (Yeow et al. 2017). *Plasmodium falciparum*-infected erythrocytes (pRBCs) have altered morphology, reduced deformability, and altered adhesive properties, whereby the pRBC can adhere to endothelial cells on blood vessel walls through the knobs on the surface of the pRBC in a process known as sequestration (Yeow et al. 2017). Sequestration enables the parasites to replicate while avoiding splenic clearance (Yeow et al. 2017). These modifications of properties in pRBCs can result in disrupted blood flow and potentially organ failure (Yeow et al. 2017). The changes in morphology of erythrocytes upon infection were imaged using AFM in a study by Millholland et al*.*, as illustrated in Fig. 3 (Millholland et al. 2011).

AFM has been used to study and compare the general changes in morphology and biomechanical properties of healthy RBCs and pRBCs according to the stages of *P. falciparum* infection or days after infection (Eaton et al. 2012). Lai et al*.* focused on the stiffening of spectrins in pRBCs, having also addressed the inhibitory effect of chloroquine on this stiffening seen in pRBCs at different stages (Lai et al. 2015).

AFM has also been used to assess potential therapeutic targets for malaria. An example of a specific new therapeutic target is the pore forming *Plasmodium* perforin-like protein (PPLP) (Garg et al. 2020). These authors designed inhibitors against pan-active MACPF Domain (PMD), a highly conserved region of PPLPs, which effectively blocked intraerythrocytic growth by suppressing the invasion and egress processes, and protected pRBCs against recombinant PMD induced senescence (Garg et al. 2020). These inhibitors also blocked the hepatic stage and transmission stage of parasite development, suggesting a multi-stage transmission-blocking option, already documented through other targets in previous studies (Garg et al. 2020; Hopp et al. 2017).

Other studies used models of diseases affecting erythrocytes that inhibit malaria infection and their potential as therapeutic targets, such as with pyruvate kinase deficiency and glucose-6-phosphate dehydrogenase deficiency (Carvalho et al. 2023; Zhang et al. 2017). For the latter, this was simulated using dehydroepiandrosterone. The effects observed include the increased stiffness of RBCs, making it harder for the parasites in the merozoite stage to invade erythrocytes, and the shrinking of cell organelles and less metabolites, suggesting that *Plasmodium* spp. multiplication would be inhibited because its organelles could not obtain enough nutrients (Zhang et al. 2017). Carvalho et al. used the treatment of erythrocytes with 2,3-diphosphoglycerate (2,3-DPG) to simulate pyruvate kinase deficiency and these erythrocytes were assessed using AFM-based SMFS, together with other methods (Carvalho et al. 2023).

AFM has also been used to clarify the mechanism of action and to monitor the therapeutic effect for known antimalarial drugs, including chloroquine and forms of artemisinin, as well as novel delivery systems to avoid the systemic side effects of these drugs (Ma et al. 2021; Olafson et al. 2015, 2017; Vidal-Diniz et al. 2022).

### Cardiovascular diseases

Cardiovascular diseases are a major global health burden, as well as the leading cause of mortality and specifically premature mortality: out of the 17 million premature deaths (< 70 years) due to noncommunicable diseases in 2019, 38% were caused by cardiovascular diseases (World Health Organization 2021a). AFM studies into the pathophysiology of these diseases could offer new diagnostic methods, approaches to management, and methods of risk stratification. The following sections address the use of AFM in the context of some of the most frequent and significant cardiovascular diseases.

#### Heart failure

Heart failure (HF) is a clinical syndrome characterized by the progressive inability of the heart to pump enough blood for the body requirements. It has been estimated that this condition currently affects over 64 million people worldwide, having a great impact in terms of mortality and morbidity, and being the end stage for cardiovascular disease for many before death (Savarese et al. 2023).

AFM was used to determine certain aspects of pathophysiology including contributing factors to endothelial and cardiomyocyte stiffness, myocardial extracellular matrix, and contractility. Much research aimed to study biological alterations responsible for diastolic dysfunction for better understanding of this process as well as to identify molecular targets, including the detyrosination of microtubules (Chen et al. 2020), changes in sarcolemmal morphology (Dague et al. 2014), the effect of intermittent hypoxia (Farré et al. 2018), and the impact of extracellular matrix remodeling (Echegaray et al. 2017; Perestrelo et al. 2021).

A novel detection method of chronic HF using AFM is based on C-reactive protein (CRP) and its anti-CRP antibody (Letchumanan et al. 2019; Yoshikawa et al. 2013). Aiming also at improving clinical prognosis, Guedes et al. assessed the changes of fibrinogen-erythrocyte interaction in patients with HF compared to control patients (Guedes et al. 2016), after the previous use of AFM to identify the receptor for fibrinogen on the surface of erythrocytes, in a study enrolling healthy blood donors and Glanzmann thrombasthenia patients (Carvalho et al. 2010). Higher fibrinogen-erythrocyte binding forces were quantified for HF patients, when compared with a healthy control group. Moreover, ischemic patients showed increased fibrinogen-erythrocyte binding forces compared with non-ischemic patients. Importantly, it was observed that higher fibrinogen-erythrocyte binding forces determined on an initial evaluation were statistically associated with a higher probability of patient hospitalization due to cardiovascular complications during the following year (Guedes et al. 2016). Therefore, the fibrinogen-erythrocyte interaction could serve as a novel risk stratification marker, pinpointing the patients at higher risk.

Novel therapeutic targets for chronic HF studied by AFM include signaling molecules to promote revascularization at the nanoscale (Hiesinger et al. 2012), the use of a nanofibrous cardiac patch to regenerate damaged cardiomyocyte tissue (Shoba et al. 2018), the inhibition of histone deacetylase (HDAC) to prevent diastolic dysfunction by enhancing myofibril relaxation (Travers et al. 2021), and the reduction of cardiac maladaptive remodeling through inhibiting cellular straining forces mediated by yes-associated protein (YAP) transcription factor (Garoffolo et al. 2022). Sigle et al. evaluated the inhibition of cyclophilin, involved in the mechanisms behind cardiac hypertrophy and remodeling present in heart failure, as a potential therapeutic target to prevent heart failure, examining the structural differences in an in vivo mouse model of angiotensin-II induced heart failure (Sigle et al*.* 2024).

Pesl et al. tested the effects of calcium, caffeine, beta-adrenergic blockers (metoprolol) and beta-adrenergic agonists (isoproterenol) on self-beating cardiomyocytes, specifically as a biosensor of cardiomyocyte contraction and relaxation (Pesl et al. 2016). These biosensors could be used more widely in clinical medicine to help characterize heart disease, among other imaging methods (Pesl et al. 2016). Chang et al. used AFM to assess the contractility of self-beating cardiomyocytes to assess the effects of inotropic and chronotropic drugs such as doxorubicin and epinephrine (Chang et al. 2013). It was proposed that this AFM model could be used to screen drugs for cardiac activity or cardiotoxicity (Chang et al. 2013).

Lyu et al. reported a simple and efficient strategy to fabricate an all-soft organoid-sensing well (Lyu et al. 2022). This AFM-like soft resistive force-sensing diaphragm, based on ultrasensitive resistive nano-cracked platinum film, offers higher sensitivity and fast responses, which are required for instantaneous detection of cardiac organoid contraction/relaxation events. Despite its simplicity, the authors show that the sensor is powerful, able to instantaneously identify key mechanical features of cardiac organoid contraction/relaxation processes under complex microenvironments. They also compare their data with the clinical presentation of diabetic cardiomyopathy, showing similar results when using the AFM-force sensing diaphragm. These data confirm that their sensor is able to detect clinically relevant pathological changes in organoid contraction/relaxation events (Lyu et al. 2022).

#### Atherosclerosis

Atherosclerosis represents the progressive stiffening of arterial blood vessel walls, which underlies several cardiovascular-related diseases. It is prevented mainly by managing cardiovascular risk factors such as arterial hypertension, smoking, dyslipidemia, and diabetes mellitus.

A significant use of AFM in investigating atherosclerosis has been to measure the change in stiffness in arterial wall cells during progression of atherosclerosis in response to factors such as location of vessels and plaques (Hayashi and Higaki 2017; Rezvani-Sharif et al. 2019a, b).

Other studies tried to focus on the impact of aging on endothelial stiffness, to account for natural changes (Berquand et al. 2021; Kohn et al. 2015, 2016). In a different approach, Dybas et al. studied the changes in erythrocytes in aging mice, compared to those with advanced atherosclerosis, with aging leading to increased erythrocyte stiffness and decreased deformability and height (Dybas et al. 2020). In terms of overall erythrocyte population, models of older mice showed erythrocytes with decreased lifespan and an increased proportion of immature erythrocytes with increased susceptibility to oxidative stress, only exacerbated in advanced atherosclerosis (Dybas et al. 2020).

**Fig. 4:**
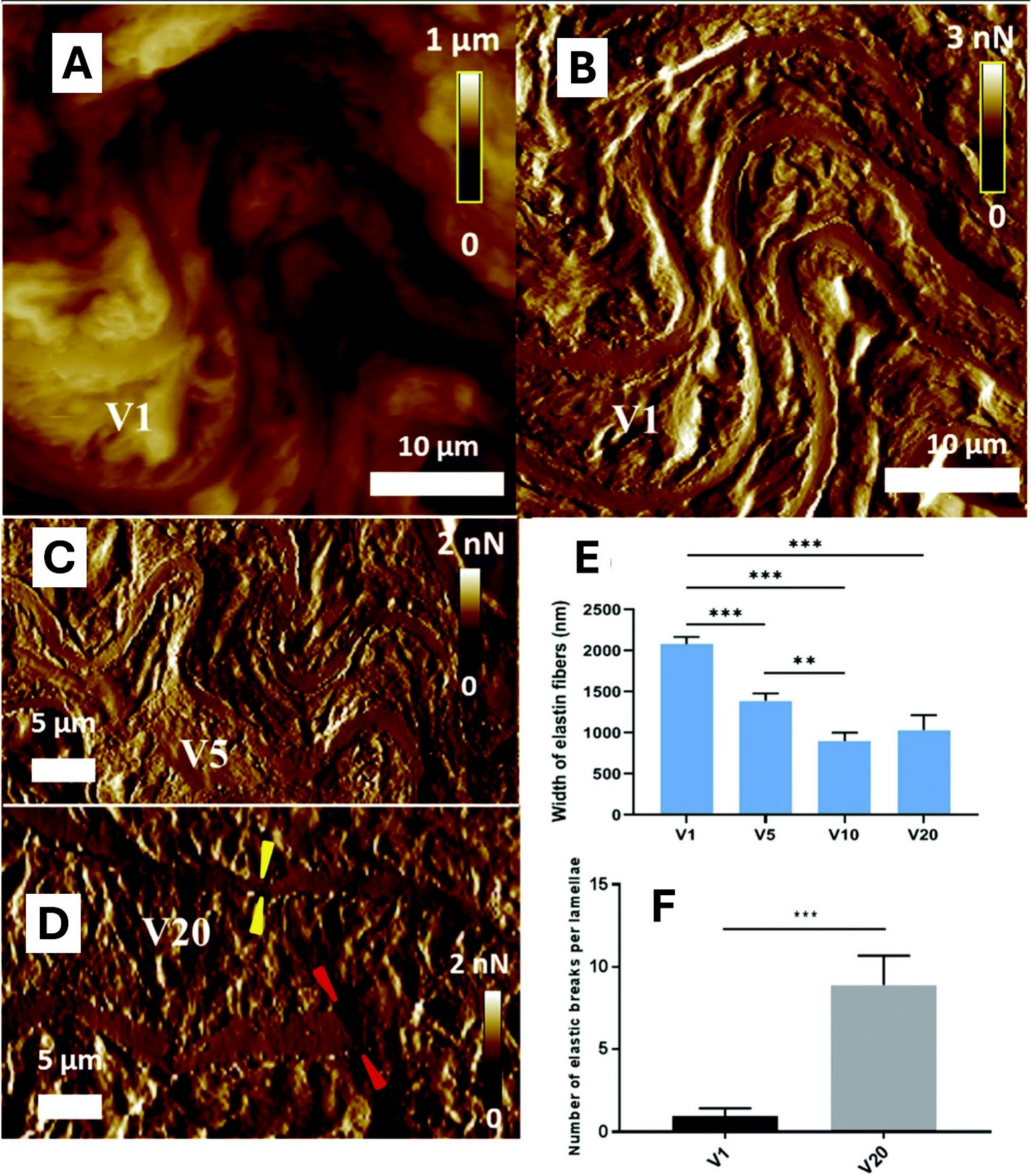
Evaluation of elastic fibers breaks and width in mice aortas during aging, using AFM analysis. **A** AFM topographical image of aorta sections from mice specimen aged 1 month (V1), and **B** associated AFM PeakForce error image. **C** AFM PeakForce error image of aorta sections from mice specimen aged 5 months (V5). **D** AFM PeakForce error image of aorta sections from mice specimen aged 20 months (V20). **E** Histogram of the width of the elastic fibers taken from PeakForce images for the different conditions including mice aged 10 months (V10). **F** Quantification of the elastic breaks per lamellae. Thick and homogeneous elastin fibers parallel to each other and very little extracellular matrix in between can be seen for V1. On the contrary, the V20 image exhibits few fibers with highly heterogeneous thickness and rupture points. Results are expressed as mean ± SEM (*n* = 15). ***, *p* < 0.001; **, *p* < 0.01 (adapted from Berquand et al. 2021, with permission)

Kohn et al. focused on the impact of aerobic exercise on atherosclerosis, showing lower macro-level stiffness for aged mice undergoing aerobic exercise, compared to those with sedentary lifestyle (Kohn et al. 2015, 2016). The high resolution of AFM used by Berquand et al. showed these age-dependent changes in model mice aged 1, 5, 10, and 20 months (Fig. 4), with the use of AFM, including an increased number of rupture points (Berquand et al. 2021). Notably, most of these significant modifications occurred in the first half of the model mice’s lifespan (Berquand et al. 2021).

Another important use of endothelial stiffness as an evaluation parameter has been the development of improved stents with vascular regeneration, since previous models showed an increased risk of thrombosis. Some approaches included the use of mesenchymal stem cells with gold nanoparticles (Hung et al. 2021), as well as the anticoagulant recombinant soluble thrombomodulin (Okamoto et al. 2020). Recently, AFM has also been used to assess the effectiveness of drugs, namely disulfiram, on slowing down atherosclerosis in a hyperlipidemic mouse model through previous studies showing its inhibition of pro-atherosclerotic signaling molecule gasdermin D, as well as modulation of phagocytosis and other atheroprotective pathways (Traughber et al. 2024).

The alteration of vascular smooth muscle cells (VSMCs) is frequently implicated in the process of atherosclerosis, being the target of other AFM studies. Some studies assessed the impact of certain factors on VSMCs, including manipulation of membrane cholesterol content (Sanyour et al. 2019), the use of statins on VSMC migration and adhesion force (Sanyour et al. 2020), the coating of type IV collagen and hyaluronic acid on stents (Li et al. 2014) and resveratrol on VSMC migration in wound healing (Lin et al. 2014).

The oxidation of low-density lipoprotein (LDL) particles is also a hallmark feature of atherosclerosis, with studies having used AFM to visualize this process and other affecting factors. The importance of oxidated LDL (oxLDL) as an early biomarker has also been studied with AFM for novel earlier detection methods for atherosclerosis, such as with acid-treated carbon nanotubes (Takeda et al. 2019), as well as cyclodextrins as a potential therapeutic option for reducing LDL cholesterol content along with its susceptibility to oxidation (Ao et al. 2016).

#### Ischemic heart disease

Ischemic heart disease (IHD) is associated with heart damage caused by the narrowing of heart arteries that supply blood to the heart muscle. It is one of the major causes of cardiovascular morbidity and mortality, with an estimated 254 million people living with ischemic heart disease, and a mortality rate of 113.94 per 100,000 in 2021 worldwide (Institute for Health Metrics and Evaluation 2024).

AFM can be a valuable tool for investigating IHD in its pathophysiology, both from the constriction of blood vessels or the death of heart muscle cells due to ischemia (Chang et al. 2023). Major components in this process are myofibroblasts and the extracellular matrix. Myofibroblasts are cells responsible for tissue repair upon inflammation and damage. However, when pathologically activated, they can promote fibrosis, lead to tissue stiffening, and replace healthy cardiomyocytes by scar tissue. The extracellular matrix is a molecular network (which includes collagen) that provides a supporting mesh to the surrounding cells in each tissue. AFM studies have focused on the increased stiffness of these components through the indentation and quantification of Young’s modulus in models of IHD or patient cells since at the macroscopic level these have been associated with diastolic dysfunction, i.e., decreased heart filling and flow to heart cells through coronary vessels (Andreu et al. 2014; Chen et al. 2020; Echegaray et al. 2017; Hara et al. 2019; Perestrelo et al. 2021). AFM studies examined components such as molecules involved in myofibroblast dysfunction-induced fibrosis (Hara et al. 2019), platelets (Li et al. 2017), extracellular matrix (Andreu et al. 2014), endothelial cells (Vahldieck et al. 2023a, b), and myocardial scar tissue (Wu et al. 2015; Yokota et al. 2020), which can then serve as novel diagnostic biomarkers or to improve prognosis. Notably, Vahldieck et al*.* studied the effect of door-to-balloon time (i.e., the time between the entrance of a heart attack patient in the emergency room and her/his cardiac catheterization) in patients with ST-elevation myocardial infarction (STEMI) vs. healthy volunteers on the nanomechanical properties of the endothelial glycocalyx, revealing a time-dependent reaction in endothelial damage and the release of pro-inflammatory mediators with a door-to-balloon time above 60 min (Vahldieck et al. 2023b).

AFM was also used to validate novel methods for earlier detection of acute coronary syndromes (ACS), using an assay specific of cardiac troponin T (cTnT) and other troponin isomers through graphene oxide, carbon nanotubes (Zhang et al. 2014), electrodes (Hasabnis and Altintas 2024; Kazemi et al. 2016; Lee et al. 2019, 2020), electropolymerization (Karimian et al. 2013), antibodies (Rodrigues et al. 2014), and surface plasmon resonance sensors (Choudhary and Altintas 2023).

The biophysical effects of therapeutic strategies on cardiomyocytes were assessed using AFM, such as sildenafil (Lee et al. 2014), a combination of GLP-1 agonists with mesenchymal stem cells (Wright et al. 2016), recombinant syndecan-1 (Vahldieck et al. 2023a, b), as well as the use of stromal cell-derived factor 1α (SDF-1α) therapy to revascularize ischemic myocardium (Hiesinger et al. 2012).

#### Arterial hypertension

Arterial hypertension is a significant condition, affecting an estimated 1.28 billion adults aged 30–79 years old, worldwide, and potentially having significant complications such as stroke, retinal disease, and kidney disease (World Health Organization 2023d). AFM may provide insights into the early detection of intermediate stages of arterial hypertension, enable a better adjustment of antihypertensive therapy before these events, or even contribute for the developing and testing of new antihypertensive drugs.

AFM has been used to detect changes in blood vessel wall morphology, as well as altered erythrocyte properties induced by arterial hypertension as an initial biomarker for future complications. Guedes et al*.* used AFM to show the increased erythrocyte-erythrocyte adhesion forces in patients with essential arterial hypertension (EAH), compared with healthy donors, as well as an increased gamma’ (*γ*’) fibrinogen (a splicing variant of fibrinogen) concentration in patients’ blood (Guedes et al. 2017). Fibrinogen, especially *γ*’ fibrinogen, can transiently promote the bridging of two erythrocytes (Guedes et al. 2017). Another study by Guedes et al. also demonstrated the higher fibrinogen-erythrocyte and erythrocyte-erythrocyte binding forces, and increased erythrocyte stiffness in a cohort of patients with EAH, which may be associated with hemorheological changes (Guedes et al. 2019). The results of these studies further established fibrinogen-erythrocyte binding as a relevant cardiovascular risk factor (Guedes et al. 2019). Another aim of AFM studies was to distinguish the effects of EAH from other comorbidities: Kaczmarksa et al. compared erythrocyte properties between healthy donors, patients with hypercholesterolemia only and patients with EAH plus hypercholesterolemia, in terms of erythrocyte topography, membrane skeleton structure, and membrane permeability to oxygen (Kaczmarska et al. 2013).

AFM was also used to evaluate the effects of known antihypertensive drugs, such as the study by Hassan et al. aimed to examine the effect of propranolol on the properties of bovine plasma fibrinogen (Hassan et al. 2011). Compared to native fibrinogen aggregates, fibrinogen-propranolol mixtures showed a higher size of the aggregate structure, with propranolol also inducing conformational changes in fibrinogen to produce a more compact surface layer, which could promote the adsorption of more molecules onto fibrinogen, with a dense packing, decreasing surface tension (Hassan et al. 2011).

### Cancer

Cancer refers to many types of disease caused by abnormal cell proliferation of different cell types. It represents another large category of global disease burden and mortality, affecting an estimated 14 million people and accounting for an estimated 9.7 million deaths worldwide in 2022 (World Health Organization 2024a). Furthermore, due to its dependence on cell mutation and chronicity, this group of diseases has become a major target for personalized medicine and therapy at the cellular level. Due to the high resolution, and ability to extract biomechanical information and assess the interaction between substances, AFM presents itself as a potential tool for diagnosis and prognosis, through the identification of the slight alterations on cancer cells, as well as a model for screening new drugs and therapeutic drug monitoring. The following sections discuss the use of AFM in breast, colorectal, melanoma, and lung cancers, due to their significant prevalence and burden in morbidity, as well as their involvement in a greater number of AFM studies.

#### Breast cancer

Breast cancer is a significant disease, especially for women: at the end of 2020, there were 7.8 million women alive who were diagnosed with breast cancer in the past 5 years (World Health Organization 2021b). AFM was used to study the morphology and characteristics of these cells, to aid in earlier diagnosis, assessment of known therapeutics, and development of new treatment methods.

AFM has been particularly useful in evaluating breast cancer cell stiffness as a parameter to distinguish them from healthy cells, as well as to compare non-invasive and invasive breast cancer cells as a novel diagnostic marker (Calzado-Martín et al. 2016; X. Chen et al. 2021; Dessard et al. 2024; M. Li et al. 2018a, b; Rother et al. 2014; Wang et al. 2016; Zbiral et al. 2023). Calzado-Martín et al*.* used AFM to correlate cell stiffness with cytoskeleton organization in healthy breast cells (MCF-10a), and cancer cells with non-invasive properties (MCF-7) and metastatic properties (MDA-MB-231) (Calzado-Martín et al. 2016). This study found that cancer cells had decreased stiffness, and a more disorganized network of actin filaments compared to healthy cells (Calzado-Martín et al. 2016).

Other studies used AFM to determine the change in intercellular adhesion forces in healthy breast cells compared to cancer cells (Abramczyk et al. 2019; Iturri et al. 2019; Kim et al. 2019; Ribeiro et al. 2016; Trache et al. 2018). Iturri et al*.* determined that cancer cells displayed a weaker cell–cell adhesion compared to cancer cells treated with anti-tumorigenic resveratrol, suggesting higher potential for cell migration and proliferation (Iturri et al. 2019; Kim et al. 2019).

AFM has not only been compared with known diagnostic tools such as elastography (Zanetti-Dällenbach et al. 2018), but also used to test novel faster prediction tests, such as surface plasmon resonance methodologies, to detect diagnostic biomarkers, including BRCA1 (Gajda-Walczak et al. 2023).

Finally, AFM has been used to examine the morphology and biomechanical properties (including adhesion and stiffness) of cancer cells to test the pro- and anti-tumorigenic effects of certain molecules. Some of these have been certain proteins expressed in cancer cells such as LRP1 (Berquand et al. 2019); P-cadherin (Ribeiro et al. 2016) and chloride channels (Yamagishi et al. 2021) with pro-tumorigenic properties and Syk protein-tyrosine kinase (Krisenko et al. 2015) and BRMS1 (McEwen et al. 2013) with anti-tumorigenic properties.

#### Colorectal cancer

Colorectal cancer is a disease with high prevalence (third most common worldwide) and high mortality, being the second most common cause of cancer death, with almost 1 million deaths per year (World Health Organization 2023a). AFM has been used to study the morphology and characteristics of these cells to aid in earlier diagnosis, the assessment of known therapeutics, and the development of new treatment methods.

AFM was used to assess the changes in morphology and biomechanical properties between healthy and colorectal cancer cells, as well as to compare the difference in these characteristics between cancer cell lines with a different motility, as prognostic biomarkers (Gavriil et al. 2023). Beton and Brozek-Pluska used AFM to examine the nanomechanical properties of normal (CCD-18Co) and cancer cell lines (Caco-2) in the gastrointestinal tract, finding increased softness in cancer cells (Beton and Brożek-Płuska 2022). Other studies compared primary cancer cell lines with lymph node metastases or peritoneal metastases (Brás et al. 2022; Lorenc et al. 2023). Another notable study was performed by Alorda-Clara et al*.*, in which bowel lavage fluid was collected from colonoscopy samples and AFM was used to characterize extracellular vesicles from tumor cells, as a source of biomarkers for the diagnosis and monitoring of colorectal cancer (Alorda-Clara et al. 2023).

**Fig. 5:**
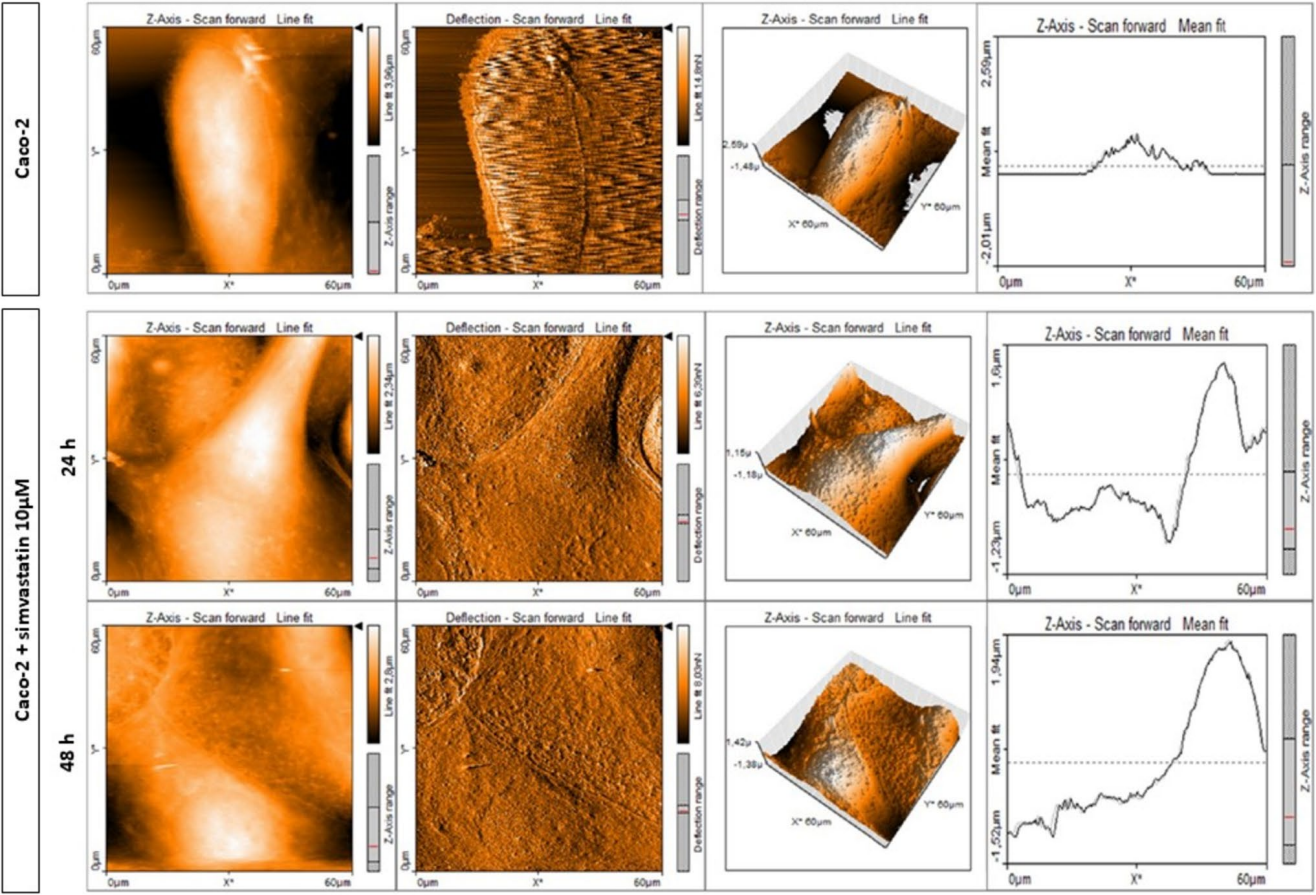
AFM topography measurements of Caco-2 cells before and after treatment with simvastatin. Topography maps of Caco-2 cells before and after supplementation with simvastatin for 24 and 48 h with deflection maps, 3D topography, and topographic sections for forward and backward traces (adapted from (Beton and Brożek-Płuska 2022), with permission)

Similar studies were conducted to assess the effects of molecules on the viability and metastatic potential of cancer cells. Some relevant molecules studied included TNF-α in triggering pro-tumorigenic epithelial-mesenchymal transition (Liu et al. 2017), zinc (Maares et al. 2020), doxorubicin (Rodríguez-Nieto et al. 2020), bevacizumab (Shen et al. 2020), and fullerenol (Liu et al. 2018). Beton and Brozek-Płuska assessed the effects of statin on nanomechanical properties of colorectal cancer cells, finding a 2.5-fold increase in cell Young’s modulus after simvastatin treatment (Beton and Brożek-Płuska 2022). The AFM images showing the changes in topography after statin treatment are displayed in Fig. 5.

#### Melanoma

Melanoma is a type of skin cancer with increasing relevance: in 2020, an estimated 325,000 new cases of melanoma were diagnosed worldwide and 57,000 people died from the disease (Arnold et al. 2022). Recent projections indicate that the incidence of melanoma per year is expected to increase by more than 50% by 2040 (Arnold et al. 2022). Since many cases of melanoma may be preventable, AFM could be used to study the morphology and characteristics of these cells to aid in earlier diagnosis, assessment of known therapeutics and development of new treatment methods.

AFM was used to examine components specific to melanoma cells with greater invasiveness, metastatic potential and response to anticancer drug treatment, through their respective alterations in biomechanical properties such as elasticity, stiffness, cell size, and cell adhesion (Brás et al. 2024; Woodcock et al. 2023). Weder et al. aimed to determine the changes in stiffness of melanoma cells during progression from non-invasive cells in radial growth phase to invasive cells in vertical growth phase and metastatic tumors (Weder et al. 2014). This study showed a decreased stiffness in the cells progressing from non-invasive compared to invasive cell lines, but a further increase in stiffness progressing towards its metastatic form. This showed that an increased ability to change cell stiffness according to their microenvironment, considered as plasticity, is more indicative of melanoma metastatic potential, unlike the uniform decrease in cell stiffness characteristic of other cancers (Weder et al. 2014). On the other hand, Makarova et al*.* aimed to use the changes in cell stiffness identified by AFM, quantified by the brush and Hertz models, to discern cancer-initiating cells compared to non-malignant melanocytes in a BRAFV600E/P53 zebrafish (Makarova et al. 2020). AFM results showed a reduced stiffness in cancer initiating cells compared to melanocytes, despite both groups having the same oncogenes.

Other studies using AFM focused on the tumor-suppressant effects of certain substances on the nanomechanical properties of melanoma cells. Some of the substances assessed included a galectin-3-binding mannan (Biscaia et al. 2022), combined treatment with colchicine/protein kinase inhibitor/myosin-II inhibitor (Luty et al. 2024) and ultraviolet radiation (UVR) (Budden et al. 2021). Budden et al. used AFM to determine the changes in dermis fibroblasts and their likelihood of melanoma formation after exposure to UVR (Budden et al. 2021). After exposure to UVR, it was shown that there was an increased extracellular matrix-degrading matrix metalloprotein-1 (MMP-1) expression and decreased expression of collagen type 1 alpha 1 chain (COL1A1), which produces type 1 collagen, and decreased invasiveness by melanoma cells (Budden et al. 2021). However, it was observed post-UVR exposure that melanoma-associated fibroblasts could restore the invasive properties of melanoma cells by increasing the production of collagen (Budden et al. 2021). Biscaia et al. found that the concomitant administration of dacarbazine decreased the number of metastatic cells and increased overall survival of melanocytes (Biscaia et al. 2022). It was also found that this anti-tumorigenic effect was reversed in melanocytes in galectin-3 (a lectin which is overexpressed in melanoma) knockout mice (Biscaia et al. 2022).

#### Lung cancer

In 2020, lung cancer was the second most diagnosed cancer (over 2.15 million cases), and remained the leading cause of cancer death, with an estimated 1.8 million deaths (almost 20% of all cancer deaths) that year (Sung et al. 2021). Given the global burden of this cancer, AFM has become important in understanding the morphology and other characteristics of these cells that could aid in earlier diagnosis and development of new treatment options.

The nanomechanical properties of lung cancer cells have been studied using AFM (Deng et al. 2018; McEwen et al. 2013; Wang et al. 2021; Zhu et al. 2021, 2022). Many of them used A549 cells as a model. McEwen et al. characterized A549 cells as having increased cell membrane roughness, as well as increased elasticity and adhesion force compared to healthy cells (McEwen et al. 2013).

**Fig. 6:**
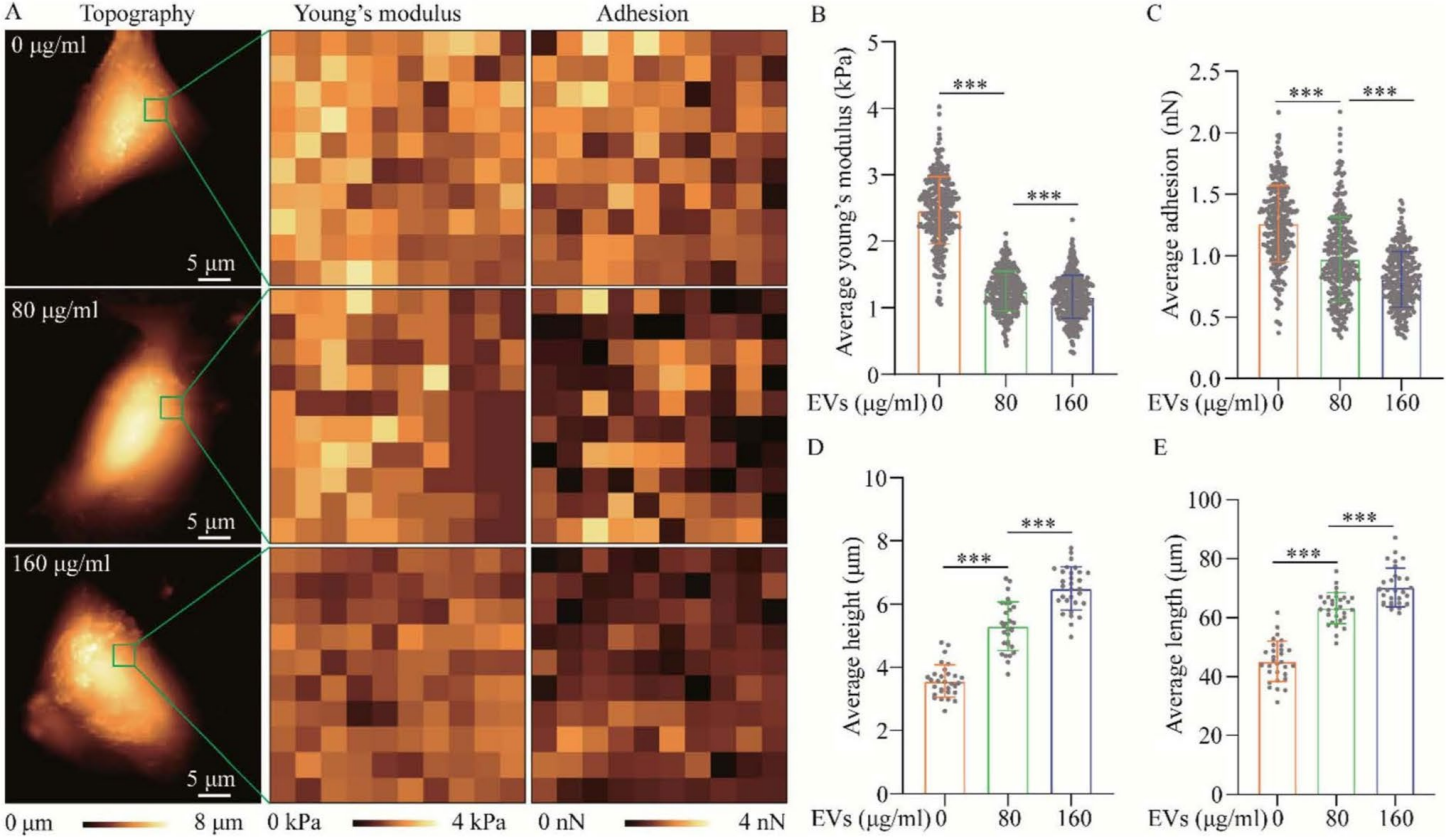
Morphology and biomechanical properties of A549 cells untreated and treated with A549-EVs for 48 h. **A** Effect of A549-EVs (at 80 and 160 μg/mL) on the morphology, Young’s modulus and surface adhesion of A549 cells, assessed by AFM. **B**–**E** Histograms of height, length, Young’s modulus, and adhesion of A549 cells with and without treatment (reproduced from (Wang et al. 2021), with permission)

Some studies aimed to discern impacting factors in the tumor microenvironment on lung cancer cells. Wang et al. aimed to study the effects of treatment of non-small cell lung cancer (NSCLC) cells with extracellular vesicles (EVs) (Wang et al. 2021). Some changes identified by AFM induced by the EVs in NSCLC cells, compared with controls, include an increased cell height and length, as well as decreased E value and cell adhesion, suggesting a more invasive phenotype (Wang et al. 2021), as shown in Fig. 6.

**Fig. 7:**
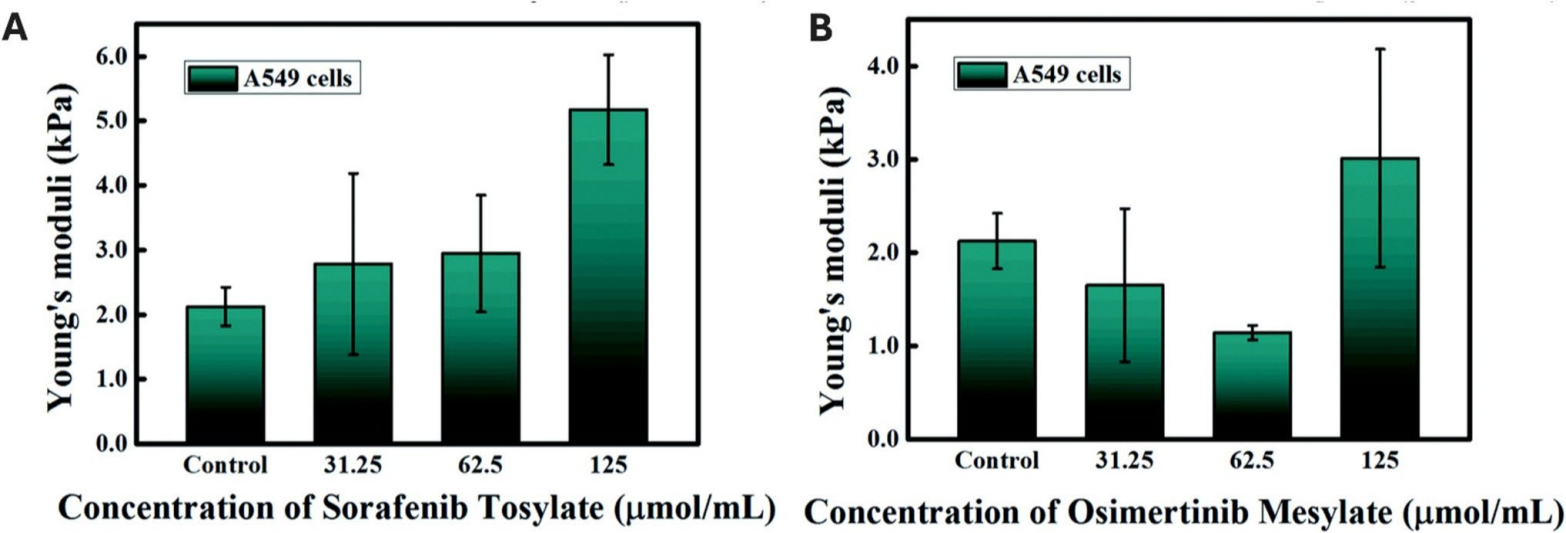
Changes in A549 cell stiffness assessed by AFM SMFS, induced by sorafenib tosylate and osimertinib mesylate. Young’s modulus after treatment of A549 cells with **A** sorafenib tosylate and **B** osimertinib mesylate (adapted from (Zhu et al. 2021), with permission)

Some other studies with AFM on NSCLC cells aimed to test new therapeutic options. Zhu et al. compared the nanomechanical effects of sorafenib tosylate and osimertinib mesylate on lung cancer cells (Zhu et al. 2021). Both drugs were found to induce anti-tumorigenic effects on lung cancer cells, including increased stiffness, as shown in Fig. 7 (Zhu et al. 2021). Further studies focused on new combined therapeutic approaches for drug-resistant lung cancer cells (Nathani et al. 2024).

### Neurodegenerative diseases

Neurodegenerative diseases are a group of diseases with significant morbidity and with potential long chronic duration, due to the increase in average longevity. There is still much unknown about the pathophysiology of these diseases, despite some having a few therapeutic options, most are aimed at symptoms. Attempts at novel medications, aimed at specific molecular targets, have been so far unsuccessful in changing the overall disease outcome. The enhanced resolution of AFM (and speed of HS-AFM) could be a potential aid in further investigation of pathophysiology and thus new therapeutic targets.

#### Alzheimer’s disease

Currently, more than 55 million people live with cognitive impairment caused by dementia worldwide, with Alzheimer’s disease (AD) being the most common form of dementia, contributing to an estimated 60–70% of cases (World Health Organization 2023b). Current therapeutic options focus on slowing down the progression of AD symptoms, namely the blocking of N-methyl-d-aspartate (NMDA) receptors by memantine. Several AFM studies have been focused on obtaining insights on the different hypotheses for AD pathophysiology, including beta-amyloid (Aβ) oligomerization and aggregation, as well as tau fibril aggregation, for diagnostic and therapeutic purposes.

AFM can be used to better understand the pathophysiology of AD, which may lead to the identification of novel therapeutic targets. As an example, Drolle et al*.* measured the interaction between neuron cell membrane and Aβ in healthy subjects, compared to AD patients (Drolle et al. 2019). They also measured changes associated with aging and changes in AD that might trigger amyloid toxicity (Drolle et al. 2019). At a macro-level, Zhao et al. used AFM to characterize the differences in stiffness of hippocampi in healthy mice, compared to mice modeling AD (Zhao et al. 2019).

Other AFM studies focused on factors that could affect AD prognosis: Menal et al*.* used AFM to assess the effect of intermittent hypoxia, edema and demyelination induced by obstructive sleep apnea syndrome (OSAS) on the risk of developing changes associated with AD (Menal et al. 2018). This study showed that it leads to decreased neuronal cell stiffness, suggestive of impaired function (Menal et al. 2018). On the other hand, Noor et al. used AFM to study the correlation between different types of fibrillation of Aβ and the development of phenotypic variants of AD in terms of classical and rapid progression of manifestations (Noor et al. 2022). Some groups used AFM to study the difference in erythrocytes, as a peripheral disease biomarker, in samples from healthy volunteers, compared to patients with AD, as well as for other neurodegenerative diseases (Nardini et al. 2022; Schneider et al. 2024; Taneva et al. 2023). The results for Nardini et al*.* showed that the time-dependent relaxation curve for viscosity and elasticity were best at distinguishing between normal and diseased erythrocytes (Nardini et al. 2022).

In terms of drug delivery, Zhang et al. used AFM to study the nano-carriers (carbon dots) containing memantine hydrochloride to specifically target tau aggregation (Zhang et al. 2022). Relating to drug development, Shao et al. used AFM to characterize the morphology of tetrahedral DNA nanostructures that inhibit the apoptosis of nerve cells (Shao et al. 2020).

#### Amyotrophic lateral sclerosis

Amyotrophic lateral sclerosis (ALS) is a degenerative motor neuron disease rarer than other neurodegenerative diseases, with global estimates of prevalence ranging from 1.9 to 6 per 100,000 people (Arthur et al. 2016). However, it is a disease with great morbidity, progressing to paralysis and respiratory failure. Due to this reserved prognosis and defined course of the disease, there has been a significant interest in better understanding this disease at the molecular level using AFM and identifying novel therapeutic targets.

Some AFM studies have been conducted to distinguish between normal and diseased variants of myocytes in ALS, based on mutations and TDP-43 protein variants. As an example, Varga et al. used AFM to examine the mechanical response of differentiated myotubes through the ALS-causing SOD1 G93A mutation, in terms of morphology and cell elasticity, detecting structural changes such as increased elasticity, which preceded symptoms and could be used as biomarker (Varga et al. 2018).

Another early diagnostic biomarker was investigated by Williams et al., which used AFM aiming at developing a detection device for disease-specific protein variants of TDP-43 through negative panning (eliminating phages that recognize the wrong variants) and positive panning (selection of phages that recognize the correct variants) (Williams et al. 2015).

**Fig. 8:**
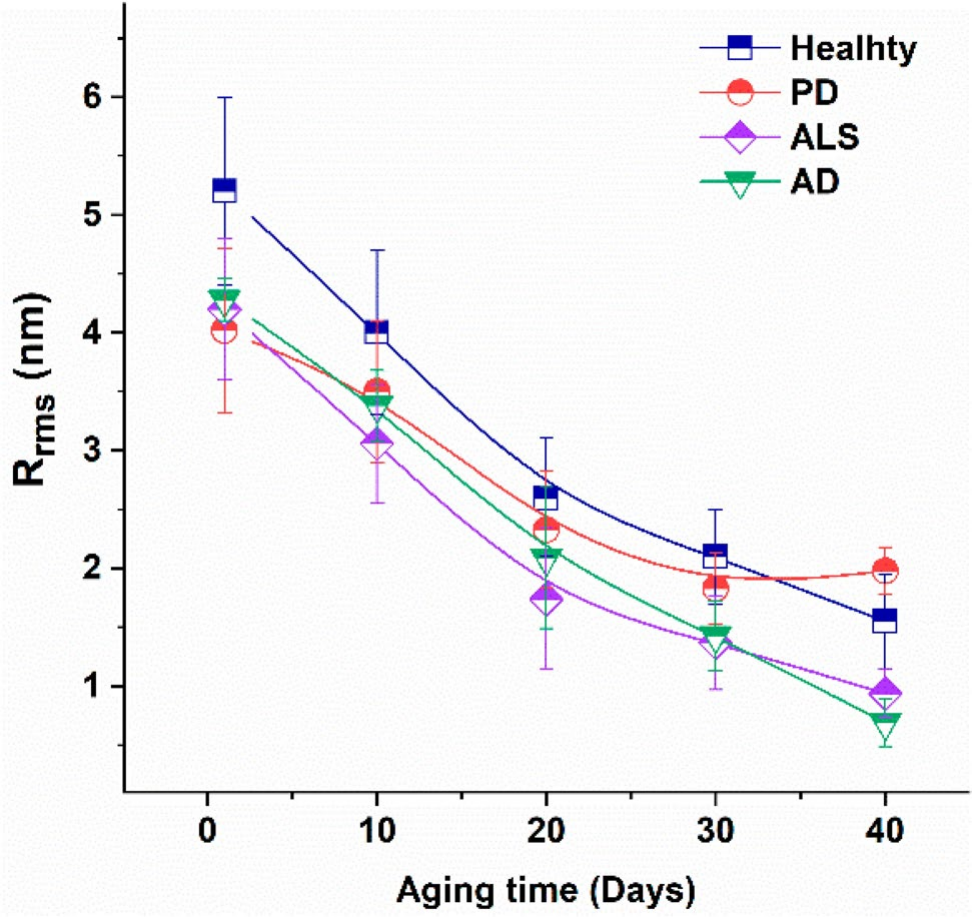
Root mean square roughness (R_rms_) of the surface of erythrocytes from healthy subjects, and PD, ALS, and AD patients vs. aging time. Mean values and Standard deviation (reproduced from Strijkova-Kenderova et al. 2022, with permission)

AFM has been used to study the changes of other cells in ALS patients apart from neurons. Strijkova-Kenderova et al*.* used AFM to compare the morphological changes in erythrocytes in healthy volunteers compared to patients with ALS, as shown in Fig. 8 (Strijkova-Kenderova et al. 2022). The same group later studied the changes in platelets in healthy volunteers, compared with neurogenerative diseases including ALS, Parkinson’s disease (PD), and AD, showing decreased surface roughness, area, and height in ALS patients (Strijkova-Kenderova et al. 2022). These two previous studies could serve as starting points for the use of blood samples as novel biomarkers and early diagnostic tools for neurodegenerative diseases (Strijkova-Kenderova et al. 2022). Lopes et al. further developed this area of study by correlating AFM nanomechanical characteristics of RBCs in ALS patients with their respective survival and functional decline, through clinical parameters, such as the revised ALS Functional Rating Scale and percentage of forced vital capacity (Lopes et al. 2024).

In terms of investigating new therapeutic strategies for ALS, Riehm et al. studied the gain in function of mutant SOD1, forming small soluble oligomers that acquire membrane toxicity, damaging membranes and “toxic channels” (Riehm et al. 2018). These misfolded SOD1 oligomers specifically target phosphatidylglycerol lipid bilayer domains, reversible in vitro by surfactant and membrane stabilizer molecule p188 (Riehm et al. 2018). This study showed that the administration of p188 on mutant SOD1 lead to delayed onset of pathological changes on motoneurons, increased survival, and decreased death of motor neurons (Riehm et al. 2018).

Another approach was used by Laos et al., which used AFM to screen drugs for amyloid-related diseases with the aim of disrupting toxic TDP-43 species before becoming longer oligomers, which increased toxicity (Laos et al. 2019). This study also used AFM to compare with current American Food and Drug Administration (FDA)-approved ALS drugs, which surprisingly were shown not to be very effective since toxic oligomers with increased length were still present (Laos et al. 2019).

#### Parkinson’s disease

Parkinson’s disease (PD) is a significant neurodegenerative disease, with estimated prevalence of over 8.5 million individuals worldwide living with it in 2019 (World Health Organization 2022b). With its progressive limitation in mobility, autonomy, and later cognitive ability, novel insights into pathophysiology and therapeutic targets with AFM have brought useful developments.

PD has been characterized by an accumulation of α-synuclein variants, which has been the target of AFM-based research to study its role in pathophysiology at the genetic level (Jiang et al. 2021), and its different phases, to assist early diagnosis.

Zhou et al. used AFM to image the change in α-synuclein oligomers at different stages of protein aggregation in terms of their toxicity and as a potential for oligomer detection and pre-symptomatic diagnosis (Zhou and Kurouski 2020). Dent et al*.* used AFM to compare the binding of α-synuclein aggregates in healthy subjects with PD patients, showing increased phosphorylation in pathologic aggregation and exploring this aggregation mechanism (Dent et al. 2022).

**Fig. 9:**
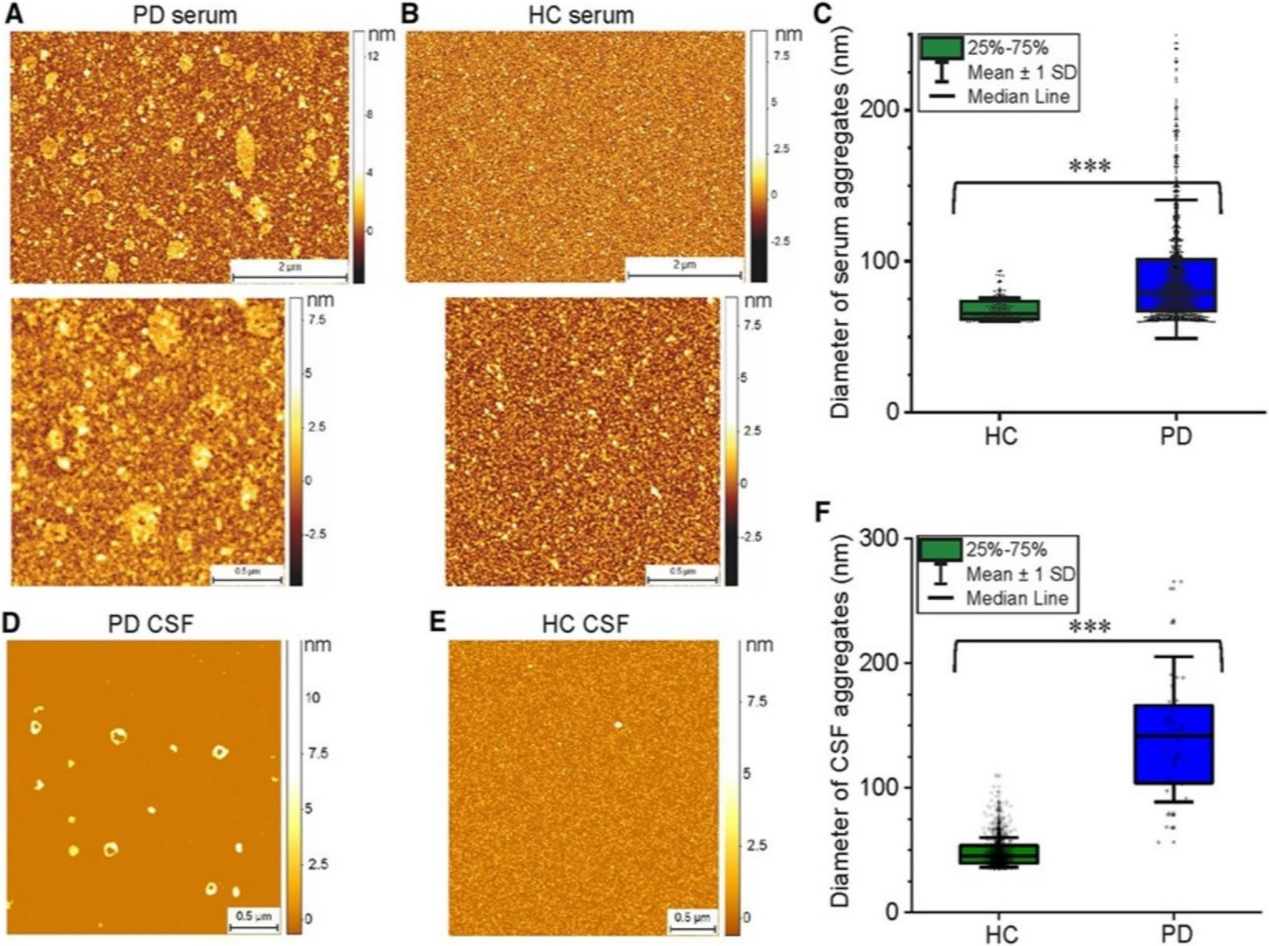
Characterization of the protein aggregates detected in PD patient serum and CSF samples, compared with healthy controls (HC), using AFM (representative AFM images of serum (**A**, **B**) and CSF (**D**, **E**) samples dried onto mica. Diameter of the aggregates (mean ± SD) detected in these serum (**C**) and CSF (**F**) samples are shown in **C** and **F** (adapted from (Lobanova et al. 2022), with permission)

The impact of the environment on α-synuclein aggregation has also been studied using AFM. Tercjak et al. used AFM to study Lewy bodies’ morphology in postmortem brain tissue, showing an aggregated fibrillary neuron structure and decreased connection between neurons (Tercjak et al. 2014). Lobanova et al*.* used AFM to characterize α-synuclein aggregates in cerebrospinal fluid (CSF) and blood of early-stage PD patients, compared to healthy volunteers, showing a difference in distribution and increased α-synuclein composition (Lobanova et al. 2022), as shown in Fig. 9.

Although PD is currently diagnosed based on clinical presentation and imaging, AFM studies have revealed potential to assess novel detection methods. For example, Li et al*.* used AFM to assess a novel detection method using skin as a sample to obtain α-synuclein using real-time quaking-induced conversion in a small group of patients (Li et al. 2024).

Other pathophysiology studies with AFM have revealed new therapeutic targets to inhibit early aberrant protein self-assembly (Ruggeri et al. 2018; Schuman et al. 2018), including DNA tetrahedrons (Yoo et al. 2019), and naturally occurring anti-α-synuclein antibodies (Braczynski et al. 2022). Another interesting study was done by Gombos et al*.,* which aimed to design drugs to destabilize bonds of insoluble amyloid deposits in β-sheet-rich structures, using the example of ferritin and magneto-ferritin (Gombos et al. 2022). The AFM results showed, on one hand, the effectiveness of ferritin in destabilizing bonds, but also the increased release of potentially harmful ferrous ions, which could cause more oxidative stress (Gombos et al. 2022).

## Conclusion

Through its progressive implementation for answering biological and clinical research questions, AFM has shown to be a promising technique for cellular-level research and personalized clinical management. Given its high resolution, different modes, combination with other microscopy techniques and greater use of high-speed versions, AFM can be further used for the evaluation of a variety of diseases. The morphological images and cell parameters obtained by AFM, as well as the cell stiffness and adhesion force quantified by SMFS, have been shown to be crucial to evaluating early steps of disease progression. This has made AFM into a key technique to develop novel early and precise diagnostic methods that may be used in clinical settings, as well in research, to investigate specific molecules and processes relevant to pathophysiology. These insights may help to identify new therapeutic targets, which can be tested on cells or biological samples in situ and in physiological conditions, providing a potential complementary approach for a faster path in drug development.

This work presents some important limitations. While this review is focused on specific clinical areas that had more robust research studies available in biomedical databases, it does not represent the totality of areas which have AFM studies published (Krawczyk-Wołoszyn et al. 2024). Furthermore, the studies in this review were mostly selected on the timeline between 2010 and 2024 and do not consider ongoing research that might represent important milestones in the mentioned clinical areas.

More research must be done using AFM in clinical settings to standardize the results obtained thus far. Additionally, clinician-researcher collaborations should be fostered to promote the clinical use of AFM. The unique qualities of this technique could further advance our understanding about human health and disease, driving future healthcare progress.

## Data Availability

No datasets were generated or analysed during the current study.
